# Historical geospatial dataset of roads and points of interest for the Chesapeake Bay Eastern Shore region of Maryland, USA, 1865

**DOI:** 10.1016/j.dib.2023.109265

**Published:** 2023-05-24

**Authors:** Jeremy Mennis, Kai Yuen

**Affiliations:** Temple University, Department of Geography and Urban Studies, 1115 W. Polett Walk, 308 Gladfelter Hall, Philadelphia, PA, USA, 19122

**Keywords:** Geospatial, GIS, Historical, Maryland, Eastern Shore, Roads, Built environment, Underground Railroad

## Abstract

The geospatial dataset presented here represents historical middle 19^th^ century built environment features for the Chesapeake Bay Eastern Shore region of Maryland, USA, including present-day Cecil, Caroline, Dorchester, Kent, Queen Anne's, Somerset, Talbot, Wicomico, and Worcester counties. Individual geospatial data layers include roads, landings, ferries, churches, shops, mills, schools, hotels, towns with post offices, and towns with court houses. These data were digitized using Simon J. Martenet's (1866) *Map of Maryland: Atlas Edition* and contemporary geospatial road network data from the Maryland Department of Transportation.

**Specifications Table**

**Table:** 

Subject	Geography
Specific subject area	Historical roads and points of interest, including settlements, shops, schools, churches, and other features
Type of data	Geospatial data; ESRI shapefile
How the data were acquired	Georeferenced digital images of historical paper maps were downloaded from a web browser-accessible repository, ingested into geographic information systems (GIS) software, and integrated with publicly-available, contemporary geospatial roads data downloaded from a state government geospatial data repository.
Data format	Raw
Description of data collection	GIS-based digitization procedures using selection, export, and manual digitization were used to extract and generate geospatial vector line and point data layers of historical road network and other settlement features embedded in georeferenced, historical map images.
Data source location	Source data were collected from: Historical map images from the David Rumsey Map Collection (https://www.davidrumsey.com/) Digital image of historical map, Caroline County, Maryland [1]. Digital image of historical map, Cecil County, Maryland [2]. Digital image of historical map, Dorchester County, Maryland [3]. Digital image of historical map, Kent County, Maryland [4]. Digital image of historical map, Queen Anne County, Maryland [5]. Digital image of historical map, Somerset County, Maryland [6]. Digital image of historical map, Talbot County, Maryland [7]. Digital image of historical map, Worcester County, Maryland [8]. Contemporary road centerlines data from the Maryland GIS Data Catalog (https://data.imap.maryland.gov/) [9]
Data accessibility	Repository name: Harvard Dataverse Data identification number: doi:10.7910/DVN/KPILKU Direct URL to data: https://doi.org/10.7910/DVN/KPILKU

## Value of the Data

• These geospatial data capture the distribution of historical 19^th^ century road networks and settlement points of interest for key features of the Chesapeake Bay Eastern Shore region of Maryland, USA. The digitization of these data from a historical atlas in paper form allows for spatial analysis of the historical distribution of settlement features in the region within geographic information system (GIS) software.
• These geospatial data may be useful for geographers, historians, economists, planners, and other researchers interested in historical patterns of transportation, development, and culture.
• These geospatial road network and settlement data were developed for the analysis of human-environment interaction in the function of the Underground Railroad in the Eastern Shore region of Maryland. However, these data can be integrated with other historical or contemporary geospatial data on changes to the natural, built, or social environments within GIS software to facilitate spatial analyses across various topical domains in the social sciences and humanities.
• These geospatial data and the methodology for generating such data from historical sources provide an example for future related research in the spatial and digital humanities.

## 1 Objective

Digital geospatial data derived from historical maps provides a key resource for historical geographic and digital humanities research [10,11], and can play a key role in understanding past inequities and injustices [12,13]. Here, we present a geospatial dataset of the locations of middle 19^th^ century roads and other built environment features for the Chesapeake Bay Eastern Shore region of Maryland, USA, as derived from Simon J. Martenet's *Map of Maryland: Atlas Edition*, published in 1866 [14]. The dataset was created to facilitate research into the historical landscapes of the Underground Railroad, a network of routes, people, and places of refuge for African Americans escaping slavery in the US South prior to the US Civil War [15]. The Eastern Shore region played a pivotal role in the Underground Railroad, as it was the birthplace and site of numerous escapes from slavery led by the famous Underground Railroad conductor Harriet Tubman. In addition, the dataset presented here may be useful for other historical analyses of the Eastern Shore region, including topics related to urban growth, economic development, transportation, and socio-cultural analyses.

## 2 Data Description

**Fig. 1:**
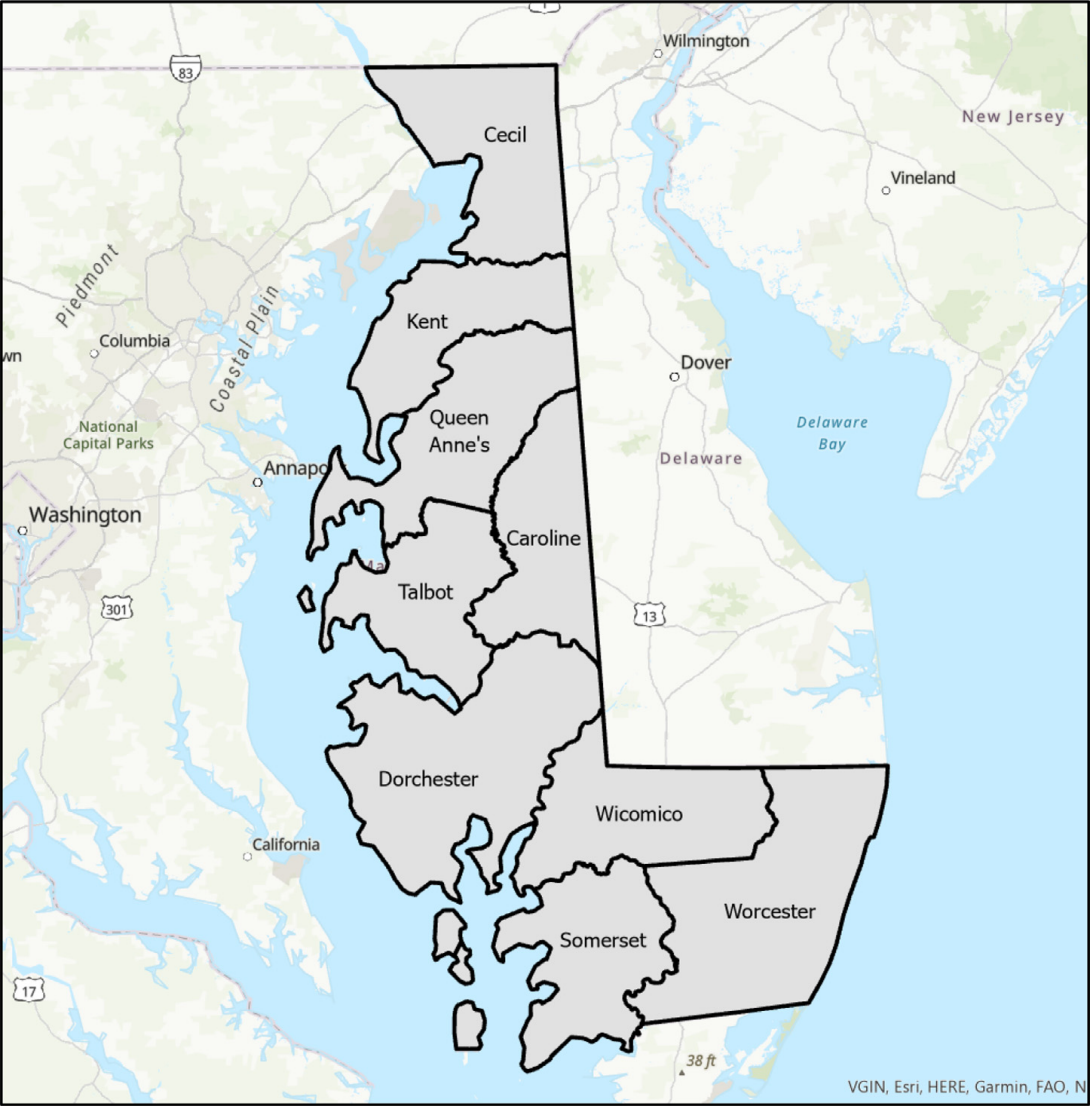
Map showing the nine present-day Maryland counties covered by the dataset. (Sources: US Census Bureau and [16] and ESRI, Inc. [17]).

**Fig. 2:**
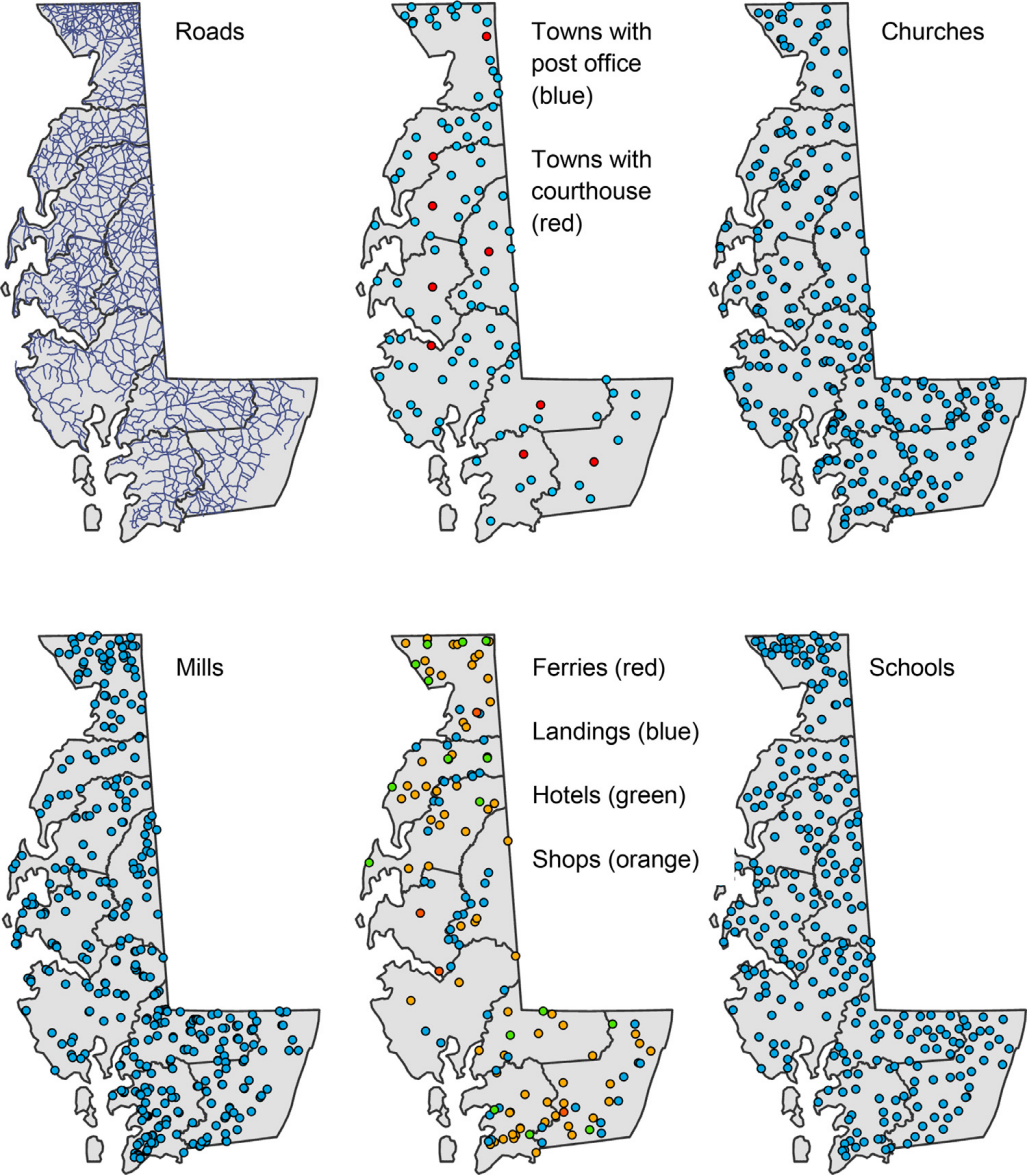
The ten shapefiles in the dataset, shown with present-day county boundaries overlain. (Sources: County boundaries from the US Census Bureau [1,16], features digitized from digital images of Simon J. Martenet's (1866) *Map of Maryland: Atlas Edition*[14] acquired from the David Rumsey Map Collection (https://www.davidrumsey.com/), and contemporary road centerlines data from the Maryland GIS Data Catalog (https://data.imap.maryland.gov/) [9]; Data Repository: https://doi.org/10.7910/DVN/KPILKU).

The dataset presented here includes a set of geospatial data layers describing the locations of historical roads and settlement features for the middle 19^th^ century Chesapeake Bay Eastern Shore region of Maryland, including present-day Cecil, Caroline, Dorchester, Kent, Queen Anne's, Somerset, Talbot, Wicomico, and Worcester counties (Fig. 1). The following geospatial layers are included in the dataset: roads, churches (including denomination, e.g. Methodist), ferries, landings (i.e. boat landings on waterways), mills (including types, e.g. saw mill), shops (including types, e.g. blacksmith), hotels, schools, towns with post offices, and towns with court houses. Fig. 2 displays maps of each of the geospatial layers overlain on the study region counties. Table 1 lists the geospatial data layers, their data type (point or line), their description, and their key attributes and domains.

**Table 1:** List of shapefiles in the dataset and their description.

Shapefile	Type	# Features	Description	Key Attributes and Value Domains
Churches	Point	272	Places of worship	Denomin (text): African American, African American Methodist, African American Meeting House, Baptist, Catholic, Episcopal, Evangelical, Methodist, Methodist Episcopal, Methodist Protestant, Protestant, Protestant Episcopal, Presbyterian, Universalist, Catholic, Quaker Meeting House, Universalist
Ferries	Point	4	Ferries	Name (text)
Hotels	Point	16	Hotels	<None>
Landings	Point	49	Boat landings on waterways	Name (text)
Mills	Point	384	Mills	Type (text): Bone, Grist, Merchant, Paper, Rolling, Saw, Steam, Wind, (can be more than one)
Roads	Line	1,196	Road network	<None>
Schools	Point	274	Schools	<None>
Shops	Point	75	Shops	Type (text): Blacksmith, Machine, Oyster House, Shoestore, Wheelwright
Towns_CH	Point	9	Named towns marked by a court house	Name (text)
Towns_PO	Point	98	Named towns marked by a post office	Name (text)

The geospatial data are in ESRI (Environmental Systems Research Institute, Inc.) shapefile format [18], a standard open source GIS format used for sharing and distributing geospatial data. Each individual shapefile is available as a .zip file [19] named by the type of feature followed by “.zip”, e.g. roads.zip. Each shapefile itself is composed of eight individual files, each named by the type of feature followed by the file type suffix, e.g. roads.shp, roads.shx, roads.dbf, and so on.

## 3 Experimental Design, Materials and Methods

### 3.1 Historical Map Source

**Fig. 3:**
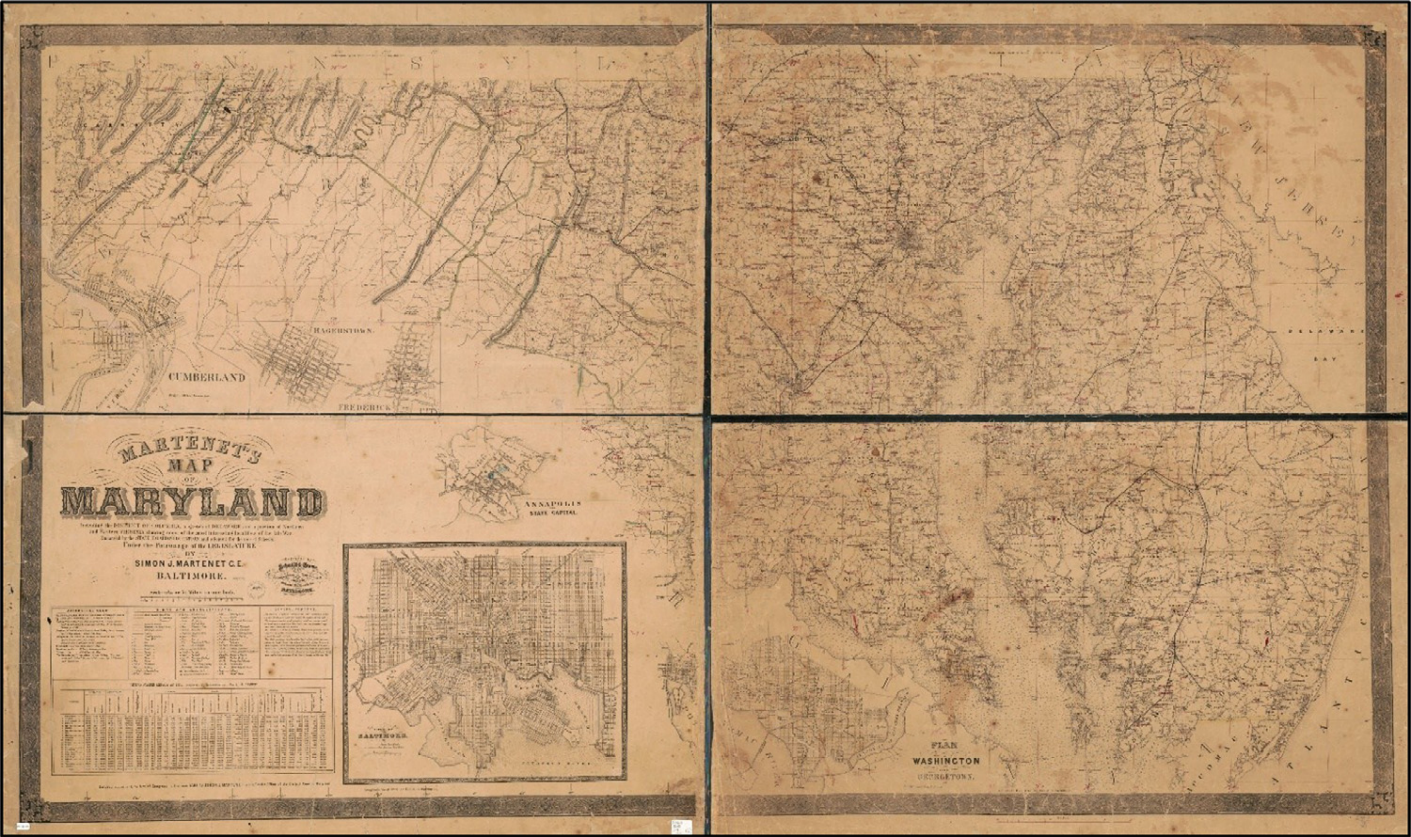
Simon J. Martenet's (1865) *Map of Maryland*[20]. (Source: David Rumsey Map Collection (https://www.davidrumsey.com/).

**Fig. 4:**
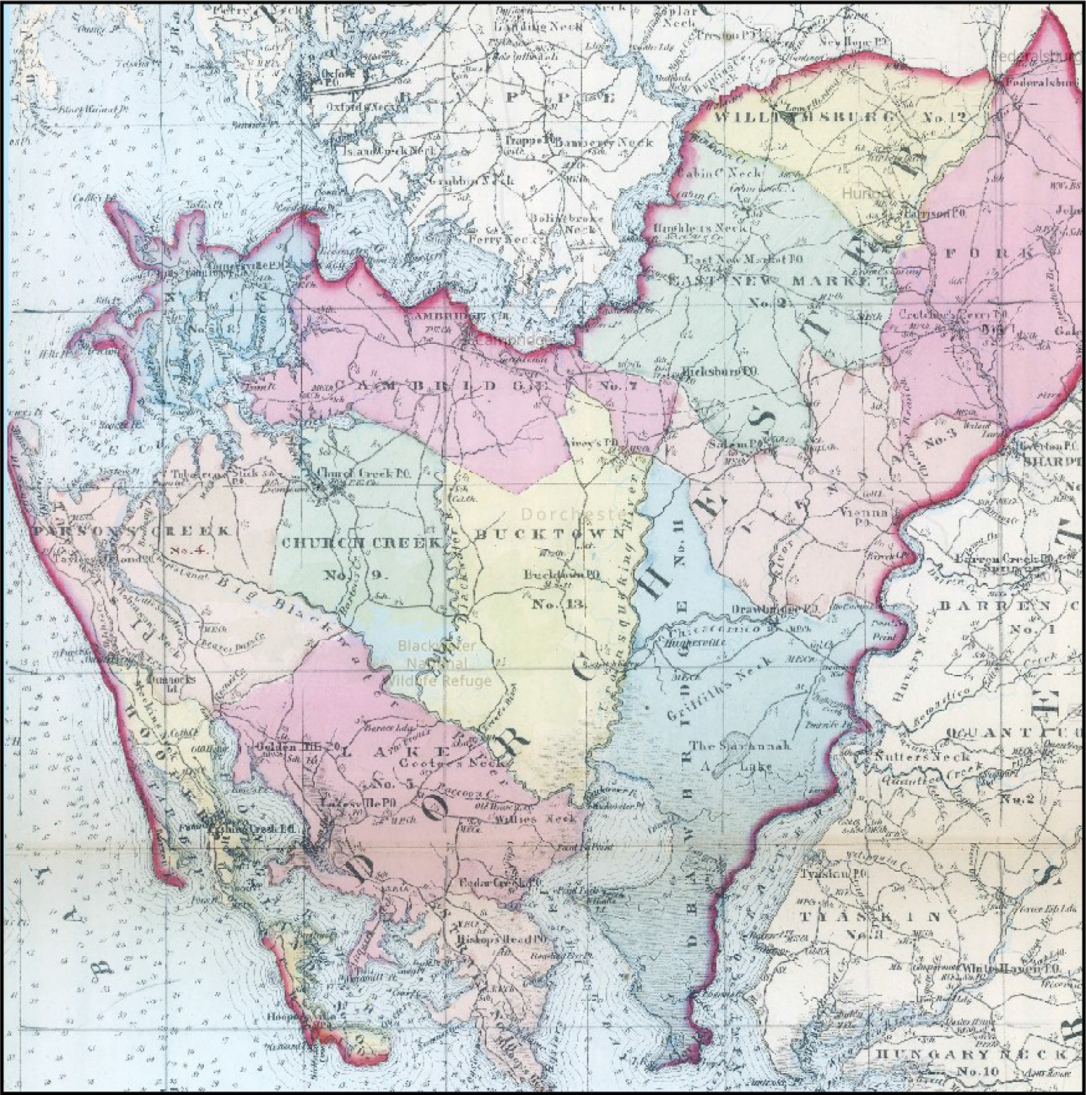
A close-up of Dorchester County, Maryland as it appears in Simon J. Martenet's (1866) *Map of Maryland: Atlas Edition*[14]. (Source: David Rumsey Map Collection (https://www.davidrumsey.com/).

The geospatial datasets were developed based on the maps contained in *Martenet's Map of Maryland: Atlas of Maryland* [14], authored by surveyor and cartographer Simon J. Martenet, which he created primarily by original surveys under the auspices of the State of Maryland. The map was first published in 1865 [20] with a scale of 1 inch = 3.5 miles (Fig. 3), then published in 1866 as an engraved color atlas in book form with each county on a separate page. Digital images of the atlas pages for the following eight historical counties were downloaded from the David Rumsey Map Collection (https://www.davidrumsey.com/): Cecil, Caroline, Dorchester, Kent, Queen Anne (also referred to as Queen Anne's), Somerset, Talbot, and Worcester counties (Table 2). Note that the contemporary Wicomico county was formed from portions of the historical Somerset and Worcester counties in 1867, following the creation of the map. Each image was downloaded as a geotiff georeferenced to the WGS 1984 Web Mercator (auxiliary sphere) coordinate system. As an example, Fig. 4 shows the image of the map for Dorchester county.

**Table 2:** List of county image files from Simon J. Martenet's (1866) *Map of Maryland: Atlas Edition*[14] used to develop the historical geospatial dataset.

County Image	File Size (kb)	Width × Height (pixels)
Cecil	33,964	4625 × 5474
Caroline	53,573	4476 × 5612
Dorchester	58,671	5385 × 8149
Kent	29,746	5124 × 4586
Somerset	60,454	5188 × 8178
Talbot	52,126	4507 × 5374
Queen Anne	50,117	5338 × 4270
Worcester	53,836	5690 × 8404

### 3.2 Contemporary Geospatial Road Centerlines Data

A digital geospatial vector line data layer in shapefile format of road centerlines for the entirety of Maryland was downloaded from the Maryland Department of Transportation through Maryland's GIS Data Catalog (https://data.imap.maryland.gov/).

### 3.3 Georeferencing and Digitizing Process

We note that georeferencing of the historical map images was provided by the David Rumsey Map Collection Georeferencer v4 [21] service hosted by OldMapsOnline [22] prior to the authors’ download of the images. Geocoding details regarding number of control points and mean positional error (MPE) were available for five of the historical county images: Caroline County (20 control points, MPE=22.3 feet), Kent County (6 control points, MPE=29.1 feet), Queen Anne County (13 control points, MPE=18.2 feet), Somerset County (9 control points, MPE=8.8 feet), and Talbot County (10 control points, MPE=18.3 feet). Georeferencing for each county image employed an affine transformation. After download, we overlaid and visually compared the georeferenced images to the contemporary road centerlines shapefile to assess evidence of systematic bias in the georeferencing (e.g. systematic directional error in feature displacement over the entire image or spatial variation across the image in positional error), but none was observed. As noted in more detail below, historical features were not digitized directly using their geographic position in the historical map image; rather, the digitizing process used to generate the geospatial dataset of historical features employed the contemporary road centerlines shapefile as a framework dataset to anchor locations of digitized features. Georeferencing quality requirements for the historical map images were thus based on the ability of the images to provide visual reference to analogous locations on the contemporary road centerlines shapefile via road shapes and intersections. Georeferencing quality was clearly sufficient for this purpose.

The digitizing process began by developing the geospatial data layer of historical roads. The vast majority of the historical roads represented in the atlas comprise a subset of Maryland's modern road network as represented in the contemporary road centerlines shapefile, and the positional accuracy of the contemporary road centerlines shapefile is clearly better than that of the historical atlas due to advances in survey technologies since the middle 19^th^ century. Therefore, the creation of the historical roads geospatial data layer proceeded by extracting the current road centerlines shapefile linework consistent with the historical roads represented in the atlas maps. This approach is similar to prior research [23] which has extracted features from contemporary digital geospatial data via overlay with georeferenced, digitized historical maps in order to maximize positional accuracy.

Using the geographic information systems (GIS) software package ArcGIS Pro (ESRI, Inc.), the current road centerlines shapefile was visually superimposed over the historical atlas map images. The individual lines in the modern road centerlines shapefile that comprised the analogous roads on the historical map were manually selected. The selected road centerlines representing the historical roads were then exported to a separate shapefile. The new historical roads shapefile was then manually edited while carefully reviewing the maps of the historical atlas to ensure accuracy, edit the shape or extent of the road linework for consistency with the historical map, manually digitize any additional historical roads not included in the contemporary road centerlines shapefile, and maintain topological integrity regarding road network connectivity (e.g. bridges over waterways in the contemporary road centerlines shapefile may not have been present historically).

**Fig. 5:**
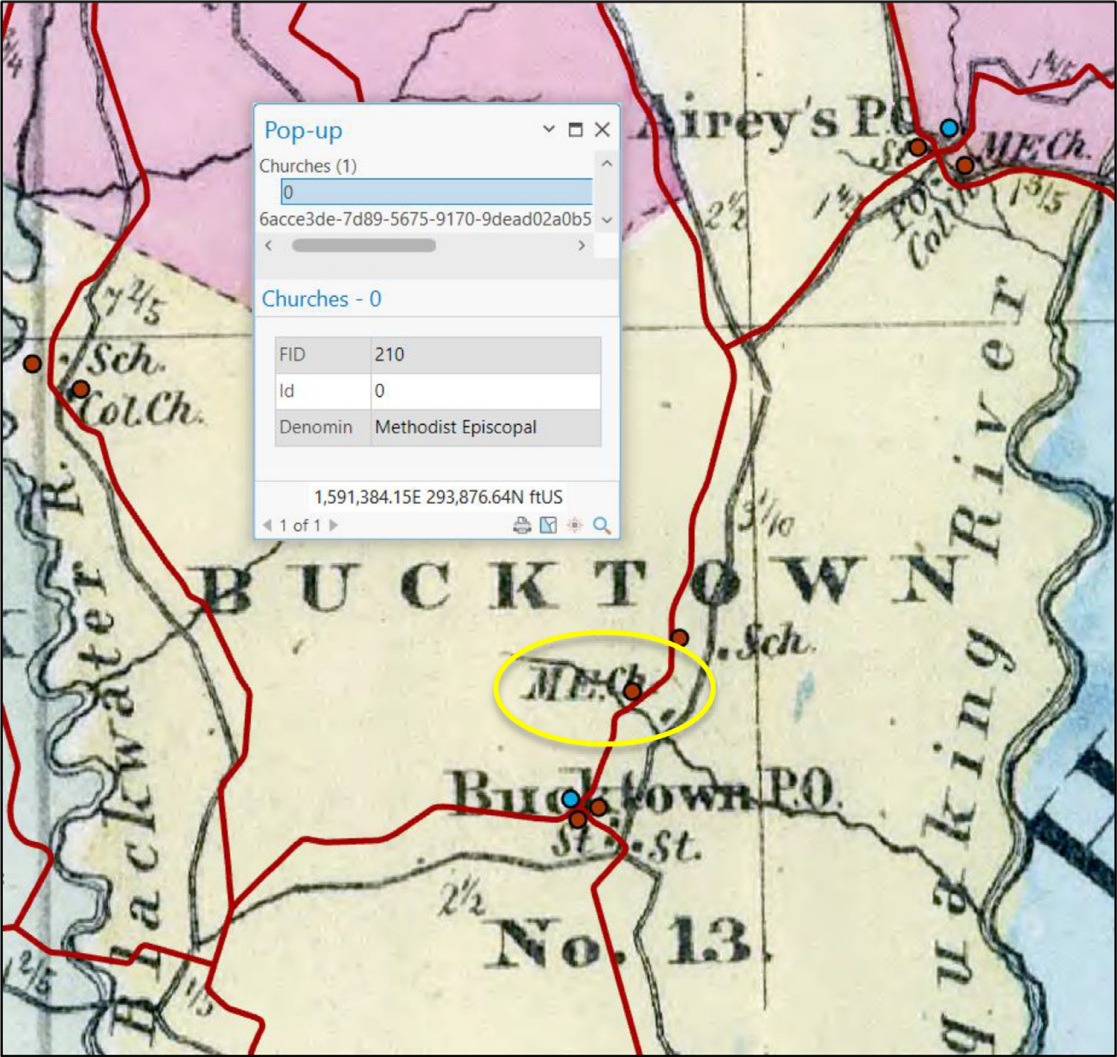
Close-up view of the historical roads shapefile (red lines) and digitized church, school, and mill features (red points), as well as town/post office features (blue points), with an associated pop-up attribute window showing the denomination for the Methodist Episcopal church circled in yellow, overlain on the historical map image of Dorchester County. (Sources: features digitized from digital image of Dorchester County appearing in Simon J. Martenet's (1866) *Map of Maryland: Atlas Edition*[14] acquired from the David Rumsey Map Collection (https://www.davidrumsey.com/), and contemporary road centerlines data from the Maryland GIS Data Catalog (https://data.imap.maryland.gov/) [9]; Data Repository: https://doi.org/10.7910/DVN/KPILKU).

**Fig. 6:**
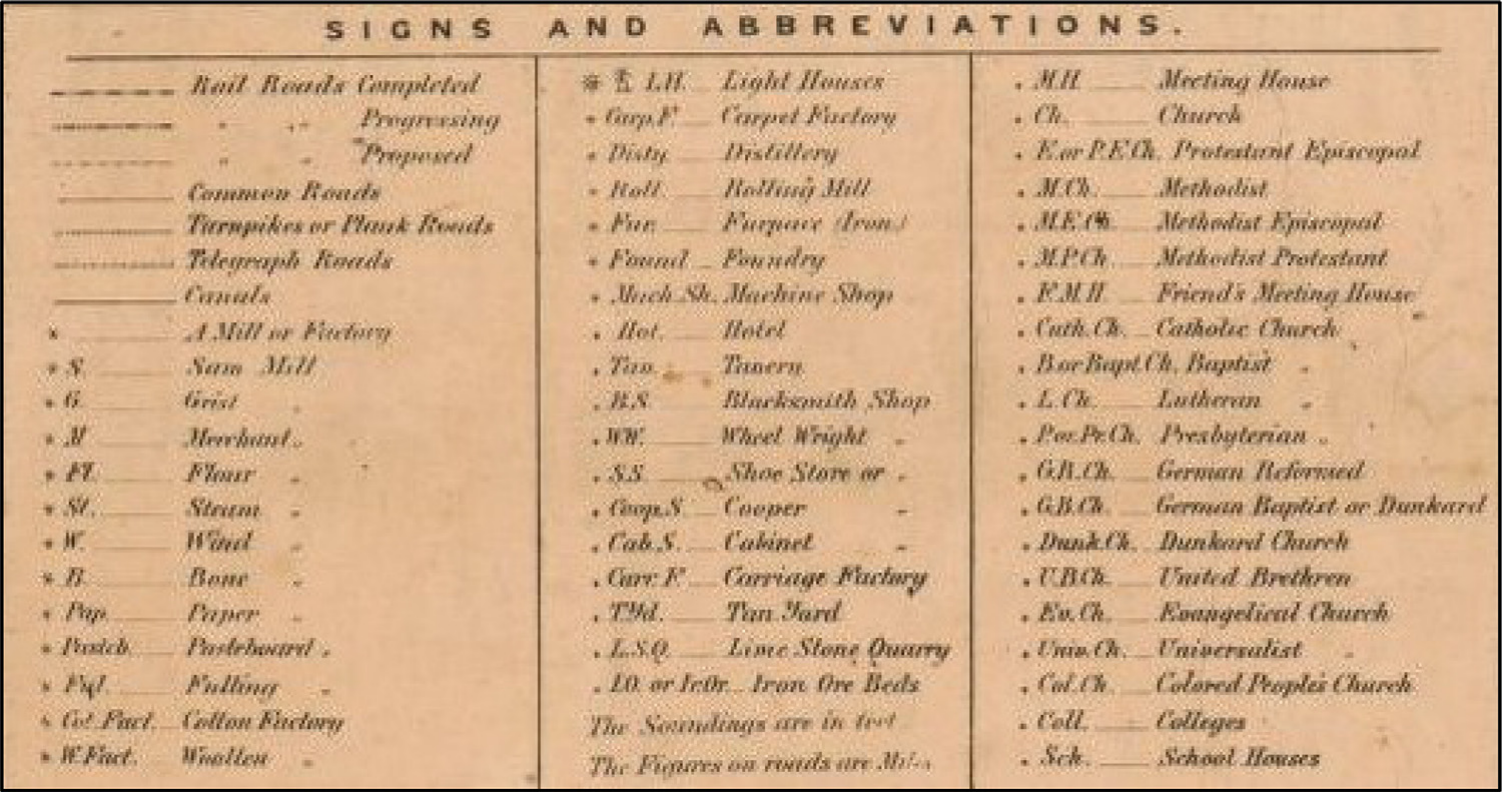
Legend in Simon J. Martenet's (1865) *Map of Maryland*[20]. (Source: David Rumsey Map Collection (https://www.davidrumsey.com/).

As with prior work generating geospatial data of historical mill locations and similar point features from paper maps [24], points of interest were digitized from the digital historical maps by digitizing directly into the GIS software using the computer screen and mouse (i.e. heads-up digitizing) [25]. Consistent with prior research that utilized contemporary digital geospatial data to georeference historical features [26], each new feature was digitized using the new historical roads shapefile to govern its relative geographic placement, as opposed to using the feature's geographic position in the georeferenced historical map image. For example, if the location of a mill occurred to the southeast of a particular road intersection on the historical map, or, say, adjacent to a visually identifiable bend in the road, it was digitized in the analogous position in relation to that road intersection or bend in the road using the new historical roads shapefile (Fig. 5). Points of interest included the following types: churches, ferries, hotels, landings, mills, shops, schools, towns with courthouses, and towns with post offices. During digitization, each point feature was attributed appropriately, based on the map legend (Fig. 6). For example, each church was attributed with the church denomination, and each shop was attributed with the type of shop (see Table 1 for all attributes and the domain of attribute values).

The historical GIS data layers are made available in the North American Datum (NAD) 1983 Maryland State Plane coordinate reference system.

### 3.4 Validation

For validation, we collected historical data from alternative sources on the county-level counts of post offices, churches, and schools in order to compare these counts to those contained in the geospatial dataset generated from Martenet's (1866) atlas. Counts of churches and public schools for 1850 were collected from the 1850 US Census via the National Historical Geographic Information System [27]. Counts of post offices at the time Martenet's (1865) map was created were collected from the historical US Post Offices dataset [28,29], which was derived from archival US Post Office Department's postmaster appointment records. Post offices which were established before 1866 and, if discontinued, were discontinued after 1866 were extracted for the Eastern Shore study region. We calculate the counts of post offices, churches, and schools for each of the eight historical counties in the study region and compare them to the analogous counts in the validation datasets.

Table 3 shows the results of the validation analysis, including counts of the post offices, churches, and schools for each county in the historical geospatial dataset (Geo) and validation datasets (Val). We tabulate the difference in counts (Val-Geo) for each county and the overall mean absolute error (MAE; $(\sum_{i=1}^{n}\left|Val_{i}-Geo_{i}\right|)/n$, where n is the number of counties) for post offices, churches, and schools. We find that counts in the geospatial dataset depart somewhat from those in the validation datasets. This is not unexpected given the data collection methodology by survey for the geospatial dataset and the historical data reporting and archival research methods used to generate the validation datasets.

**Table 3:** Validation results for post offices, churches, and schools by county (‘Geo’ indicates historical geospatial dataset, ‘Val’ indicates validation dataset, and ‘Dif’ indicates the difference in the counts [Val-Geo]; ‘MAE’ is the mean absolute error).

	Post Offices			Churches			Schools		
County	Geo	Val	Dif	Geo	Val	Dif	Geo	Val	Dif
Caroline	13	8	-5	16	21	5	25	25	0
Cecil	17	15	-2	24	39	15	44	52	8
Dorchester	20	17	-3	45	26	-19	36	33	-3
Kent	13	10	-3	17	37	20	18	29	11
Queen Anne	9	4	-5	28	23	-5	29	30	1
Somerset	12	13	1	60	57	-3	45	55	10
Talbot	5	3	-2	21	28	7	26	30	4
Worcester	8	5	-3	59	60	1	50	49	-1
MAE			3.0			9.4			4.8

With the exception of Somerset county, each of the counties in the geospatial dataset contain a greater number of post offices as compared to the validation dataset, with an average difference of three post offices per county. We speculate that some of the more remote or rural post offices may have served informally or were not tracked in official national US post office records of postmasters. The geospatial dataset tended to undercount schools and churches as compared to US Census Bureau records, particularly in Kent county. There are many more schools and churches than post offices and the MAE for schools and churches is higher as compared to post offices. Potential reasons for the disparity between the counts for the historical geospatial and validation datasets are that Martenet's survey simply did not identify all the schools and churches in the region or may also be due to changes which occurred between the survey and 1850 census data collection.

## Ethics Statements

This research did not employ human or animal studies. All data used in this research was publicly available and did not require special permission for use.

## CRediT authorship contribution statement

**Jeremy Mennis:** Conceptualization, Methodology, Writing – original draft, Data curation. **Kai Yuen:** Data curation, Writing – review & editing.

## Declaration of Competing Interest

The authors declare that they have no known competing financial interests or personal relationships that could have appeared to influence the work reported in this paper.

## Data Availability

Geospatial Dataset of Roads and Settlement Features for the Chesapeake Bay Eastern Shore Region of Maryland, USA, 1865 (Reference data) (Dataverse).
